# Yeast-Based Aβ1-15 Vaccine Elicits Strong Immunogenicity and Attenuates Neuropathology and Cognitive Deficits in Alzheimer’s Disease Transgenic Mice

**DOI:** 10.3390/vaccines8030351

**Published:** 2020-07-01

**Authors:** Dong-qun Liu, Shuai Lu, Lun Zhang, Ya-ru Huang, Mei Ji, Xiao-ying Sun, Xiao-ge Liu, Rui-tian Liu

**Affiliations:** 1 National Key Laboratory of Biochemical Engineering, Institute of Process Engineering, Chinese Academy of Sciences, Beijing 100190, China; dqliu@ipe.ac.cn (D.-q.L.); slu@ipe.ac.cn (S.L.); zhanglun@ipe.ac.cn (L.Z.); yrhuang@ipe.ac.cn (Y.-r.H.); jimei@ipe.ac.cn (M.J.); xysun@ipe.ac.cn (X.-y.S.); xgliu@ipe.ac.cn (X.-g.L.); 2 School of Chemical Engineering, University of Chinese Academy of Science, Beijing 100049, China

**Keywords:** Alzheimer’s disease, beta-amyloid, active immunotherapy, yeast, vaccine

## Abstract

Immunotherapy focusing on reducing the amyloid-beta (Aβ) burden is a promising treatment strategy for Alzheimer’s disease (AD). Many clinical studies on AD immunotherapies have failed due to low safety and efficacy, calling for a highly potent AD vaccine which induces sufficient antibody titer while avoiding side effects. Here, we designed a yeast-based vaccine Y-5A15 comprising five copies of Aβ1-15 displayed on the surface of yeast cell wall, and we subcutaneously immunized APP/PS1 mice three times. Our results demonstrated that the Y-5A15 remarkably enhanced the Aβ epitope immunogenicity and elicited high antibody titers against Aβ in AD mice. Importantly, Y-5A15 vaccination successfully reduced Aβ levels, plaque burden and glial activation, rescued synaptic deficits and significantly ameliorated memory and cognitive decline in APP/PS1 transgenic mice, suggesting that the yeast-based Aβ epitope vaccine has a promising potency for the treatment of AD.

## 1. Introduction

The neuropathologies of Alzheimer’s disease (AD) are characterized by deposition of β-amyloid (Aβ) peptide in senile plaques and the formation of intracellular neurofibrillary tangles (NFT) [1,2]. Aβ peptides have been confirmed to play a central role in the onset and progression of AD for over 20 years, and the soluble Aβ oligomers and protofibrils, which are now considered to be the most toxic forms of Aβ, responsible for synaptic destruction [3,4,5,6]. Anti-Aβ immunotherapy is an efficient strategy to relieve the Aβ burden, which has been verified in various AD transgenic models. Unfortunately, the first vaccine clinical trial in AD patients (AN-1792) failed due to meningoencephalitis in 6% of immunized AD patients, which was associated with autoreactive T cell infiltration into the brains of immunized subjects in combination with a strong, Th1-based adjuvant, QS21 [7,8,9]. Another major issue with the AN-1792 vaccine was insufficient antibody titer due to the weak immunogenicity of Aβ, which limited the therapeutic effect [10,11,12].

Notably, the dose-dependent effect of anti-Aβ oligomer/protofibril antibodies on Aβ burden and cognition was recently demonstrated in patients with mild AD [7,10,13]. An expanded dataset from the phase 3 EMERGE trial on patients receiving high-dose aducanumab reported statistically significant changes in the Clinical Dementia Rating-Sum of Boxes (CDR-SB) scores [14], suggesting that a potential Aβ-based vaccine should be administered preventatively and induce robust antibody titer to halt or at least delay the development of AD pathologies. To avoid autoreactive T cells against Aβ and to generate relatively high titers of antibodies, the secondary generation of Aβ vaccine was developed by conjugating B-cell epitopes, N terminus of Aβ42 to foreign carriers to overcome low immunogenicity of B-cell epitopes [15,16,17,18]. However, none of the above AD vaccines has produced clinically meaningful results, calling for a more optimal vaccine platform for the future development of Aβ epitope vaccines.

The yeast cell is a promising vaccine carrier and adjuvant which can be naturally recognized by APCs and has been approved by the US Food and Drug Administration (FDA) for clinical test, generally recognized as safe (GRAS) by FDA [19,20]. Some studies have shown that recombinant EBY100 *Saccharomyces cerevisiae* is an effective vaccine carrier, which expresses antigens on the cell wall through a yeast display system [21,22]. Yeast using a surface display system can express high-density heterologous proteins, is easily prepared, extremely stable and low cost [23,24]. Moreover, the epitopes displayed on the surface of particles can provide the most efficient antigen presentation to B cells, which is benefit for inducing rapid and robust antibody response [25]. Thus, the yeast cell is considered to be a superior vaccine development and antibody induction platform.

In order to induce sufficient antibody titer against Aβ and efficient improvement on cognitive capacity, we developed Y-5A15 yeast cell as a vaccine carrier and immune adjuvant, comprising five copies of Aβ1-15 displayed on the yeast cell wall, and assessed its therapeutic effect on APP/PS1 mice.

## 2. Materials and Methods

### 2.1. Materials

EBY-100 S. cerevisiae strain was kindly provided by Dr. Xiang-mei Liu, Shandong University, Jinan, China. The Aβ42 and Aβ40 immunoassay kits were purchased from Immuno-Biological Laboratories Co., Ltd. (Gunma, Japan). 6E10 (monoclonal antibody against Aβ1-16; BioLegend, San Diego, CA, USA), 4G8 (monoclonal antibody against Aβ17–24; Signet Laboratories/Covance Research Products, Denver, PA, USA), 9E10 (anti-c-Myc antibody, Santa Cruz, CA, USA), Iba-1 polyclonal antibody (Genetex, Irvine, CA, USA), and GFAP monoclonal antibody (Cell Signaling Technology, Danvers, MA, USA). HRP-conjugated goat anti-mouse IgG antibody and FITC-conjugated goat anti-rabbit IgG antibody were obtained from Beijing Zhongshan Golden Bridge Biotechnology Co., Ltd. (Beijing, China).

### 2.2. Preparation of Yeast-Based Vaccine

To enhance the immunogenicity of the epitope peptides, the gene fragment encoding the five copies of Aβ1-15 fragment was inserted into a modified vector of pCTCON2 and transfected into EBY-100 (*Saccharomyces cerevisiae*) using the method described previously [26]. The epitopes displayed on the yeast cells were then assayed by flow cytometry and confocal microscopy after probed with FITC-labelled anti-c-Myc antibody. The EBY100 carrying the vehicle vector was the negative control. The EBY-100 cell-based vaccine was heat-inactivated for 1 h at 56 °C and stored at −80 °C until use.

### 2.3. Mouse Immunization

Six-month-old APP/PS1 transgenic AD mice were obtained from Jackson Laboratories (BarHarbor, ME, USA). The transgenic mice were randomly assigned to treatment with yeast vector control YEVC (*n* = 6), Y-5A15 (*n* = 6), or AD control (*n* = 6), and their WT littermates (*n* = 6) were used as a positive control for the behavior test. The mice were immunized 3 times with 6 × 10^7^ cells in biweekly intervals. Blood was collected in regular intervals and stored at −80 °C until use. The effects of Y-5A15 on the cognition of AD mice were detected 20 days after the last administration. All experimental protocols were approved by the Tsinghua University Animal Care and Use Committee and were performed in accordance with the China Public Health Service Guide for the Care and Use of Laboratory Animals.

### 2.4. Antibody Titer Determination

The titers of vaccine-induced antibodies in mouse sera against Aβ42 peptide were determined by indirect enzyme-linked immunosorbent assay (ELISA). Briefly, 96-well ELISA plates were coated with Aβ42 peptide (0.5 μg/well) at 4 °C overnight and blocked with 3% (*w*/*v*) bovine serum albumin in PBS for 2 h at 37 °C. After blocking, 2-fold serial dilution of sera were added in triplicates and incubated for 2 h at 37 °C. The bound serum antibodies were detected with HRP-conjugated anti-mouse IgG and TMB substrate. The reaction was stopped by adding 2 M H_2_SO_4_, and the absorbance of OD450 nm were read on microplate reader MD-SpectraMax M5 (Molecular Device, Sunnyvale, CA, USA).

The levels of different IgG isotypes were also assayed by ELISA with the HRP-conjugated anti-mouse -IgG1, IgG3, -IgG2a and -IgG2b antibodies as the secondary antibodies (Abcam, Cambridge, UK), respectively.

### 2.5. Novel Object Recognition (NOR) Test

Mice have the spontaneous tendency to exhibit more interactions with a novel rather than a familiar object. Each mouse was put in a white box at 40 × 40 × 40 cm and allowed to freely explore the open field arena without objects in the habituation phase. Each mouse was then put in the box containing two identical objects for another 5 min during the familiarization period. Recognition memory was assayed after 24 h by exposing the mouse to one familiar and one novel object. The exploring and sniffing times on each object were recorded.

### 2.6. Y-Maze Test

The Y-maze test was executed to assess short-term memory capacity of mice. Y-mazes consist of three arms with an angle of 120° between each of the two arms. The three identical arms were randomly designated: the start arm kept open, in which the mouse was put to start exploring, the novel arm was open just during the 2 nd trial but blocked during the 1st trial, and the other arm was always kept open. To assess the spatial recognition memory, the Y-maze test had two trials separated by 1 h of inter-trial interval (ITI). The first trial (training) had a 10 min duration and allowed the mouse to explore the start arm and the other arm, with the third arm (novel arm) being blocked. After 1 h ITI, the mice were placed back to the maze in the same starting arm, with free access to all three arms for 5 min during the second trial. All trials were recorded on a videocassette recorder using a ceiling-mounted charge-coupled device (CCD) camera, and the number of entries and time spent in each arm in the video recordings were analyzed.

### 2.7. Morris Water Maze (MWM) Test

The MWM test was performed in a 1.2-m-diameter white iron pool at 22 ± 1 °C during 5 training days. The mice were released into the pool at one designated start location for each trial and were allowed to find a platform hidden 1–2 cm under the water surface. Each training lasted 60 s until to find hidden platform. All the mice were allowed to stay on the platform for 10 s. The swimming activity of each mouse was recorded by an automated overhead-mounted video tracking system (Sony Corp., Tokyo, Japan). On day 6, the platform was removed from the pool and the mouse were allowed to explore the pool for 60 s, and then the escape latencies, the time in the target quadrant and the number of target crossings were measured.

### 2.8. Cerebral Homogenate Collection

After the behavioral tests, all mice were deeply anesthetized and perfused with ice-cold PBS containing 10 U/mL heparin. Half brains were dissected and rapidly homogenized. The brain tissues were bounce homogenized in solubilization buffer containing protease inhibitor cocktail (50 mM Tris, pH 7.4, 150 mM NaCl, 2 mM EDTA, 1% sodium deoxycholate, 1% Triton X-100), then centrifuged at 14,000× *g* for 30 min at 4 °C. The supernatant was used to assess soluble Aβ. The insoluble pellets were homogenized with 2% SDS buffer (50 mM Tris, pH 7.6), and centrifuged at 14,000× *g* for 1 h at 4 °C to obtain supernatants containing insoluble Aβ.

### 2.9. Measurement of Aβ40/42

To detect soluble and insoluble Aβ levels in the brains, an anti-human Aβ_1-42_ and Aβ_1-40_ ELISA kits were used according to the manufacturer’s instructions. The levels of soluble and insoluble Aβ were standardized to the brain tissue weight.

### 2.10. Immunohistochemistry (IHC)

Immunohistochemical staining was performed as previously described [27]. Briefly, brain cryosections (20-μm thick) were treated with 80% (*v*/*v*) methanol containing 0.3% H_2_O_2_ to prevent endogenous peroxidation. After blocking with 10% goat serum, the sections were incubated with primary antibodies 4G8 (1:100), 6E10 (1:100), Iba-1 (1:100) or GFAP (1:100) for 1 h at room temperature (RT), and then incubated with a fluorescent-labeled secondary antibody at RT for 30 min. Images were collected on an Olympus BX60 microscope (Olympus Optical Co., Ltd., Tokyo, Japan) or confocal scanning laser microscope (LSM780, Zeiss, Oberkochen, Germany), respectively. For analysis of the images, we used IpWin5 analysis software.

### 2.11. Statistical Analysis

The data were expressed as mean ± SD. The data was processed and analyzed with GraphPad Prism 6.01. ELISAs data were performed at least three times for each experiment, and six mice per group were analyzed for behavior tests and IHC analysis. Data were analyzed using one-way ANOVA or Student’s *t*-test. Differences were considered significant at * *p* < 0.05, ** *p* < 0.01, and *** *p* < 0.001.

## 3. Results

### 3.1. The Preparation of Y-5A15 Vaccine

**Figure 1:**
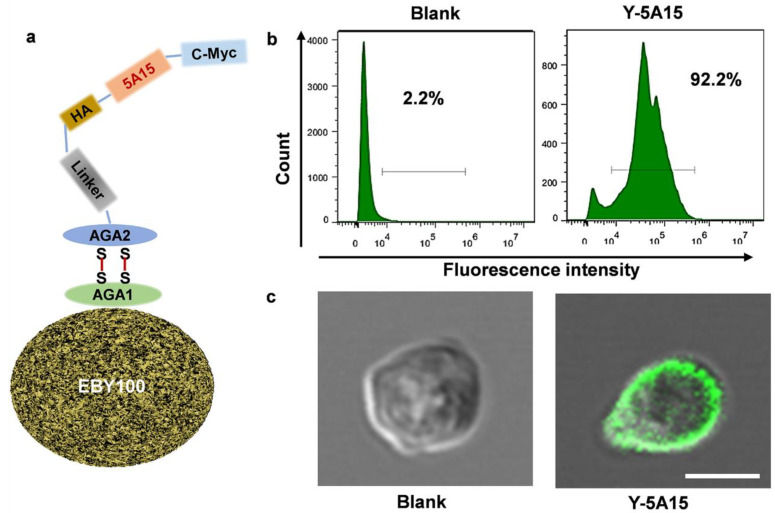
The construction and characterization of the Aβ1-15 epitope on the EBY-100 yeast cell wall surface. (**a**) Schematic illustration of the construction of yeast-based vaccine. The epitope antigen with c-myc were displayed on the surface of cell wall by AGA2 interaction with AGA1. (**b**) The 5 × Aβ1–15 epitopes expressed on the surface the EBY100 yeast cells were testified by flow cytometry using anti-c-Myc antibody and FITC-labeled secondary antibody. EBY100 yeast cells were used as a negative control. (**c**) The 5 × Aβ1–15 epitopes in (**b**) were determined by confocal microscopy. Bar indicates 2 μm.

To induce maximal antibody titer and avoid the activation of autoreactive T-cells, we used five copies of Aβ1-15 as the antigen and the EBY100 *Saccharomyces cerevisiae* cells as the vaccine platform to develop a novel AD vaccine. The epitope was inserted between HA and c-Myc tags with a GSG linker, which enabled all the Aβ1-15 antigens to be presented on the surface of the recombinant *Saccharomyces cerevisiae* yeast (Figure 1a).

To further test the exhibition of 5 × Aβ1-15 epitope on the surface, the yeast cells were incubated with anti-c-Myc antibody, then incubated with secondary antibody labelled with FITC, and detected by flow cytometer and confocal microscope, respectively. More than 90% Y-5A15 yeast cells showed positive signal in comparison to EBY100 alone (Figure 1b). Moreover, the confocal results confirmed that 5 × Aβ1-15 peptides were displayed on the surface of the yeast cells, while no green fluorescence was detected on the EBY100 cell control (Figure 1c).

### 3.2. Y-5A15 Vaccine Effectively Elicits High-Titer Antibodies against Aβ42 in AD Mice

**Figure 2:**
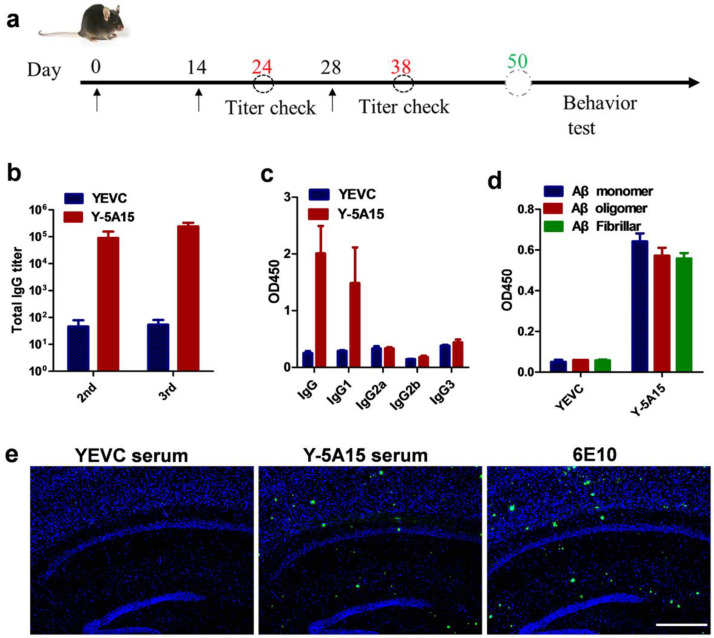
The anti-Aβ response of plasma antibody induced by Y-5A15 vaccine. (**a**) Schematic diagram of vaccine immunization for APP/PS1 mice (*n* = 6). (**b**) Aβ_42_ antibody levels induced by Y-5A15 vaccine. (**c**) The subtypes of vaccine-induced antibodies against Aβ_42_. (**d**) The binding of antibodies induced by Y-5A15 vaccine to different Aβ isoforms was detected by ELISA. (**e**) Mice AD brain sections were stained using serum from Y-5A15-vaccinated APP/PS1 mice. Brain tissue was detected via IHC using serum from YEVC (1:200)- or Y-5A15 (1:200)-treated mice. Scale bar is 500 μm.

APP/PS1 mice were immunized subcutaneously with 6 × 10^7^ Y-5A15 yeast cells for three times in the absence of adjuvant, and the serum antibody titer was detected by ELISA (Figure 2a). The results demonstrated that the Y-5A15 vaccine but not yeast vehicle (YEVC) induced a robust antibody response in mice (Figure 2b). The antibody titers increased with the immunization times and reached a plateau after the third vaccination (Figure 2b). Moreover, the antibody induced by YEVC had high affinity for Aβ monomer, oligomer and fibrillar (Figure 2d). The isotypes of the antibodies in response to Aβ42 vaccine were detected by ELISA. Y-5A15 preferentially induced IgG1 antibody isotype (Figure 2c), indicating that predominant Th2 immune response was involved in Y-5A15 immunization.

To further detect the immune response, we performed IHC using antibody 6E10, the sera from Y-5A15 vaccine, as well as sera from YEVC-treated mice. The results showed that 6E10 and the sera induced by Y-5A15 vaccine bound to Aβ plaques in the brains of APP/PS1 mice, whereas no positive signal was observed with the addition of the sera from YEVC-treated mice, indicating that the induced antibodies recognized Aβ plaques (Figure 2e).

### 3.3. Y-5A15 Vaccination Attenuates Cognitive Impairment in APP/PS1 Mice

**Figure 3:**
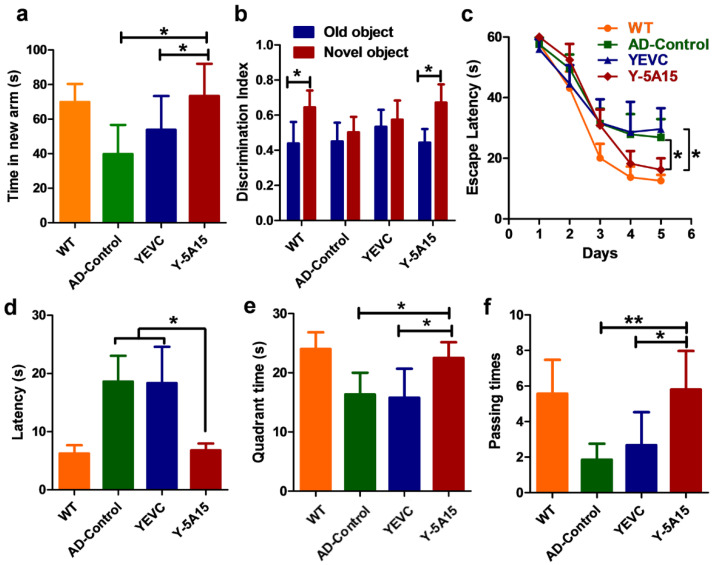
Y-5A15 vaccination improved cognitive capacity in APP/PS1 transgenic mice. (**a**) In the Y-maze, the time spent by mice in the new arm. (**b**) Discrimination index of AD mice tested by novel object recognition (NOR). (**c**) The escape latency in the acquisition period of the Morris water maze (MWM). (**d**) The escape latency in the probe trial of the MWM without the platform. (**e**) The target quadrant time in the MWM probe test. (**f**) The times of crossing the platform location in the MWM probe test. One-way ANOVA with LSD was used for statistical analysis. *n* = 6 mice per group, * *p* < 0.05, ** *p* < 0.01.

To evaluate the effect of Y-5A15 immunization on the cognition of APP/PS1 mice, we conducted behavioral tests, including spontaneous Y-maze, NOR and MWM tests. Compared with the YEVC-treated mice, Y-5A15-immunized mice spent more time in the new arm (*p* < 0.05) during the Y-maze test, indicating that Y-5A15 improved short-term memory in APP/PS1 mice (Figure 3a). During the probe trial of NOR test, the exploration times of mice treated with Y-5A15 for the novel object were significantly increased compared with the familiar object (*p* < 0.05), while the AD control and YEVC-treated mice exhibited similar exploration times on both novel and familiar objects (Figure 3b).

The MWM test was performed to detect the effects of Y-5A15 immunization on the spatial memory and learning ability of APP/PS1 mice. During the training phase of the test, the mice were given 1 min to search for the hidden platform under surface for 5 days. Compared with the AD control and YEVC-treated APP/PS1 mice, mice treated with Y-5A15 readily found the location of the hidden platform after 3 days of training (*p* < 0.05) (Figure 3c). After the last training, the mice were put in the maze to find the location of the removed platform for 1 min. Y-5A15-treated mice exhibited spatially oriented swimming behavior and shorter escape latencies (Figure 3d) and increased number of target crossings (Figure 3f), and they spent more time in the target quadrant (Figure 3e), indicating that Y-5A15 substantially improved the spatial memory and learning ability of APP/PS1 transgenic mice.

### 3.4. Y-5A15 Vaccination Reduces Cerebral Aβ Levels in AD Transgenic Mice

**Figure 4:**
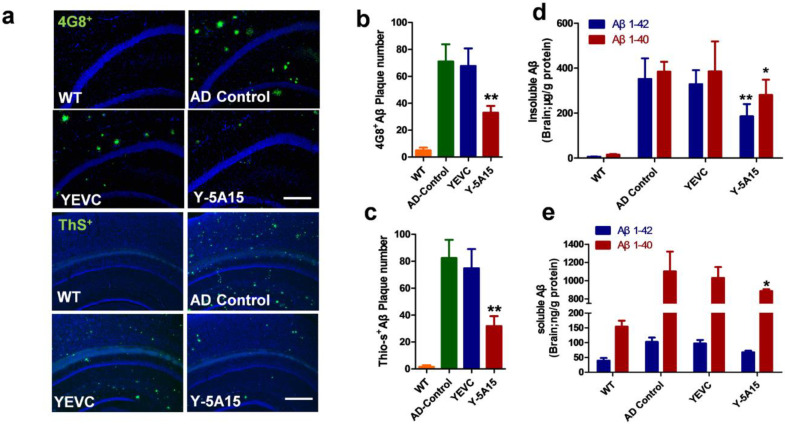
Y-5A15 immunization decreased cerebral amyloid-β (Aβ) peptide levels in the brains of APP/PS1 transgenic mice. (**a**) Representative microscopic images of brain sections from different groups. Sections were stained with the anti-human Aβ antibody 4G8 (Green) and thioflavin S (ThS), counterstained with DAPI (blue) to detect nuclei. The number of 4G8-positive (**b**) and ThS-positive plaques (**c**) were quantified in the hippocampus (Hip) and cortex (Ctx) by IpWin5 software. (**d**) ELISA analyses of insoluble human Aβ40 and Aβ42 in the brains. (**e**) ELISA analyses of soluble human Aβ40 and Aβ42 in the brains. Compared with AD control, * *p* < 0.05, ** *p* < 0.01, Student’s *t*-test, *n* = 6 mice per group. Scale bar is 200 μm.

To study the effects of Y-5A15 vaccine on brain pathologies in AD mice, an initial evaluation of Aβ plaque burden was assessed in the cortex and hippocampus of mice (Figure 4a–c). Brain sections were immunolabelled with anti-human Aβ monoclonal antibody (mAb) 4G8 or fluorescently labelled with thioflavin-S (Thio-S) to assess diffused and mature/fibrillar Aβ plaque load, respectively. The results showed that 4G8^+^ Aβ plaque burden substantially reduced in the hippocampus of Y-5A15-treated mice but not in the YEVC-treated and AD control mice (Figure 4a). Moreover, in the brains of mice immunized with Y-5A15, the number of Thio-S^+^ Aβ plaque deposition also significantly relieved (Figure 4a). Quantitative analysis of Aβ plaque load on serial brain slices covering the hippocampus and cortex showed that compared with the AD control groups, Y-5A15-treated mice showed remarkable reduction in the levels of 4G8^+^ and Thio-S^+^ plaque by 53% and 60%, respectively (*p* < 0.01) (Figure 4b,c), indicating that Y-5A15 vaccination conspicuously alleviated the Aβ plaque load in the brain of APP/PS1 mice.

The levels of insoluble Aβ_40_ and Aβ_42_ in the brains of mice immunized with Y-5A15 significantly decreased by 27% and 47%, respectively (Figure 4d,e). The Y-5A15 vaccine effectively relieved the level of Aβ_40_ by 21% in soluble brain extracts (Figure 4e, Red bars), but has no significant effect on the level of soluble Aβ_42_ (Figure 4e, Blue bars). Taken together, these results demonstrated that Y-5A15 vaccination significantly reduced the amount of plaque deposition and levels of Aβ_40_ and Aβ_42_ in the brain of APP/PS1 mice.

### 3.5. Y-5A15 Vaccination Suppresses Astrogliosis and Microgliosis in the Brains of AD Mice

**Figure 5:**
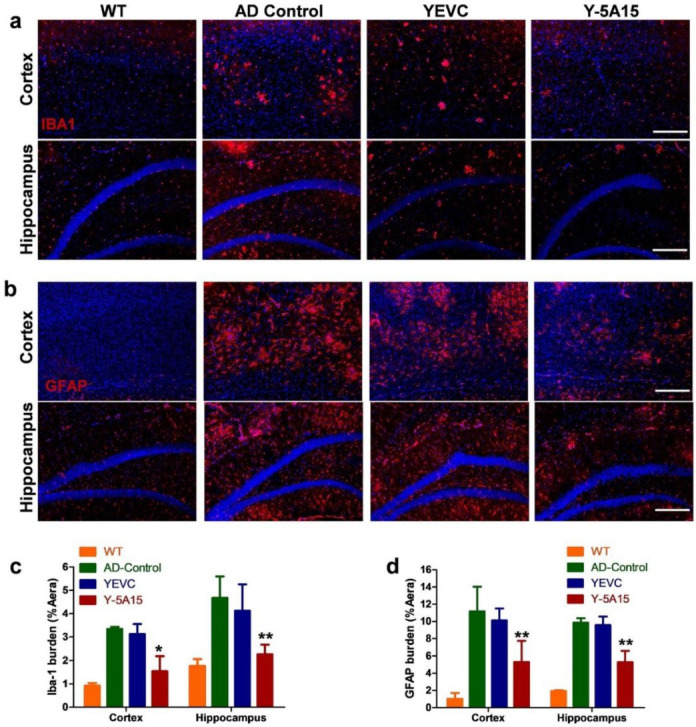
Y-5A15 vaccination decreased activated microglia and astrocytes in the hippocampus and cortex of APP/PS1 transgenic mice. (**a**) Representative fluorescence micrographs in the cortex and hippocampus of brain slices detected by staining Iba-1, and then the stained microglia were quantified using IpWin5 software (**c**). (**b**) Astrocytes in the cortex and hippocampus of brain slices were determined by staining GFAP, and then the stained astrocytes were quantified using IpWin5 software (**d**). Compared with AD control, * *p* < 0.05, ** *p* < 0.01, Student’s *t*-test, *n* = 6 mice per group. Scale bar is 200 μm.

In AD, microglia and astrocytes accumulate at Aβ plaque lesion sites and exhibit increased reactivity and cytotoxicity. Overactivation of glial cells may be involved in vascular disease in mixed dementia and even in “pure” AD pathology [28]. To address whether vaccine treatment was effective for astrogliosis and microgliosis in the brains of APP/PS1 transgenic mice, we stained astrocytes and microglia with antibodies against Iba-1 and GFAP, respectively. The results showed that Y-5A15 vaccination reduced microgliosis especially in the cortical and hippocampal regions (Figure 5).

Quantitative immunohistochemical analyses of Iba1^+^ and GFAP^+^ cells’ total area were performed in the cortex and hippocampal regions from each experimental group. Y-5A15 strongly reduced mean cortical and hippocampal Iba1^+^ cell area by 54% and 52%, respectively (Figure 5c). Moreover, Y-5A15 treatment remarkably reduced mean cortical and hippocampal GFAP^+^ cell area by 53% and 46%, respectively (Figure 5d). These results suggested that Y-5A15 treatment significantly suppressed astrogliosis and microgliosis in AD mouse brain, which may also benefit for vascular cognitive impairment acting as a disease-modifying molecule [29].

### 3.6. Active Immunotherapy by Y-5A15 Yeast Vaccine Rescues Synaptic Deficits in APP/PS1 Mice

**Figure 6:**
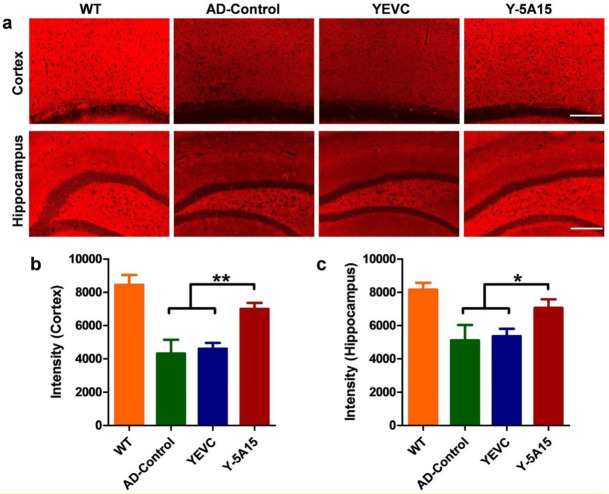
Active immunotherapy using Y-5A15 vaccine rescued synaptic deficits in APP/PS1 transgenic mice. (**a**) Presynapses in brains of mice were detected by IHC with anti-synaptophysin antibody in various immunized groups. Synaptophysin in the cortex and the hippocampal regions (**b**,**c**) was quantified using IPwin5 Image-Pro Plus software, respectively, and results are shown in arbitrary units (AU). * *p* < 0.05, ** *p* < 0.01. Scale bar is 200 μm.

The Aβ accumulation and accompanying neuronal loss have been corroborated in the previous studies [30,31]. To assess whether the reduction of Aβ accumulation can attenuate synaptic deficits, we used anti-synaptophysin antibody to detect the effect of Y-5A15 yeast vaccine on the synapses. AD mice vaccinated with Y-5A15 showed significantly higher synaptophysin densities in the cortex and hippocampal regions compared with control groups (Figure 6), indicating that Y-5A15 yeast immunization rescued Aβ-induced synaptic deficits in APP/PS1 mice.

## 4. Discussion

We here designed and prepared a *Saccharomyces cerevisiae*-based AD vaccine, Y-5A15, and provided strong evidences that Y-5A15 yeast vaccine could serve as effective antigen carrier and adjuvant to enhance the immunogenicity of Aβ1-15, and induced rapid antibody production, exerting favorable effects on cognition and neuropathology in the APP/PS1 mice.

In order to improve immunogenicity of the short Aβ1–15 peptide, we constructed a five-valent foldable Aβ1–15 in the design of Y-5A15 yeast vaccine, with a GSG small linker separating each peptide sequence (5 × Aβ15). Consistent with the previous report that S. cerevisiae yeast expressed high copies of the protein scaffold-Aga2 fusions [23], the 5 × Aβ1-15 peptides were densely displayed on the yeast cell wall and over 90% Y-5A15 yeast cells expressed antigen. All AD mice immunized with Y-5A15 yeast vaccine developed sufficient Aβ antibody titers. Our further results demonstrated that the induced Aβ antibody had a high affinity to Aβ monomer, oligomers and fibers, indicating that Y-5A15 vaccine may reduce insoluble Aβ or Aβ deposits as well as soluble forms of Aβ, including the neurotoxic oligomers which impair synaptic and cognitive function [32]. Importantly, the predominant antibody induced by Y-5A15 vaccine was IgG1 isotype, indicating that the immune response to the vaccine was mainly involved in the Th2 phenotype and that yeast-derived carrier may be a safe vaccine platform.

High Aβ antibody titers induced by Y-5A15 vaccine attenuated cognitive impairment in APP/PS1 mice. NOR, Y-maze and Morris water maze tests were performed to evaluate the efficiency of Y-5A15 vaccination in improving cognitive function in APP/PS1. Y-5A15-treated mice demonstrated a significant improvement in novel object exploration, short-term memory and spatial memory. Fibrillar Aβ plaques have been demonstrated to correlate with microglyosis and astrocyosis in AD transgenic mouse models and patients [33,34,35]. In the study, the Aβ plaques were attenuated in the immunized APP/PS1 mice, which was consistent with the decrease of insoluble Aβ extracted from the brain. These results may further induce fewer Iba-1-immunopositive microglia and GFAP-immunopositive astrocytes. Moreover, Y-5A15-vaccinated APP/PS1 mice had higher synaptic density, which may contribute to their superior cognitive performance compared to the control AD mice. Like other vaccines using yeast as a carrier, Y-5A15 yeast vaccine was characterized by easy preparation, good safety, and low cost [36], suggesting that Y-5A15 held promising potential for AD treatment. However, before the translation of Y-5A15 into clinical application, its acute and long-term toxicity, long term stability, pharmacokinetics, and the duration of the induced antibody in peripheral blood should be detected.

## 5. Conclusions

In conclusion, our study indicated that the yeast-based Y-5A15 vaccine exerted favorable effects on cognition and neuropathology in the APP/PS1 mice by inducing high titers of antibodies against Aβ, decreasing Aβ burden, suppressing microgliosis and astrogliosis in mouse brains, and increasing synaptophysin levels. These findings suggest that Y-5A15 may be a safe and effective vaccine for the treatment of AD.
